# Pacemaker Reel Syndrome Presenting as Recurrent Syncope: A Case Report

**DOI:** 10.7759/cureus.104576

**Published:** 2026-03-02

**Authors:** Neha Chopra, Rakavi Rathinasamy, Shrividya Rao, Anwar Ansari, Devesh Kumar

**Affiliations:** 1 Cardiology, Hero Dayanand Medical College (DMC) Heart Institute, Ludhiana, IND; 2 Cardiology, All India Institute of Medical Sciences, New Delhi, IND; 3 Cardiothoracic Surgery, Max Super Speciality Hospital, New Delhi, IND; 4 Cardiology, Vardhman Mahavir Medical College and Safdarjung Hospital, New Delhi, IND

**Keywords:** complete heart block, complications of pacemaker implantation, lead dislodgement, pacemaker lead displacement, reel syndrome

## Abstract

An elderly woman presented with recurrent syncope for two weeks. She had undergone a single-chamber permanent pacemaker implantation (PPI) two months ago. At presentation 12-lead electrocardiogram revealed complete heart block. Cinefluoroscopy revealed that the right ventricular (RV) lead had dislodged and was lying in the superior vena cava. On comparison with prior cinefluoroscopy, the pulse generator appeared to have rotated about its transverse axis, with leads coiled around the generator, consistent with Reel syndrome. The right-sided pocket was re-explored. The lead was seen to be coiled on top of the pacemaker generator. Right axillary venous access was taken, and an active fixation lead was implanted in the RV. She was discharged with instructions regarding careful arm movement.

## Introduction

Cardiac implantable electronic devices (CIEDs) are implanted worldwide in increasing numbers [1]. CIED complications are more frequent than acknowledged, with most studies reporting risks of 5-6% for any complication and 3-4% for major complications after implantation [2]. We present a case of an elderly lady, post-permanent pacemaker implantation two months back, who presented to us with recurrent syncope for two weeks. Evaluation revealed lead dislodgement with rotation of the pulse generator around its transverse axis, consistent with Reel syndrome. The following sections review lead macro-dislodgement syndromes and their management.

## Case presentation

An elderly, obese lady in her 80s presented to the emergency department with recurrent syncope for two weeks. She had been diagnosed with complete heart block with wide escape rhythm two months ago, for which she had undergone right-sided single-chamber permanent pacemaker implantation at another center. She also had gradually progressive memory impairment for the last five years. On examination, her heart rate was 40 beats per minute, blood pressure 150/90 mmHg, and oxygen saturation 96%. The respiratory and abdominal examinations were non-contributory, and she had no focal motor or sensory deficits.

**Figure 1:**
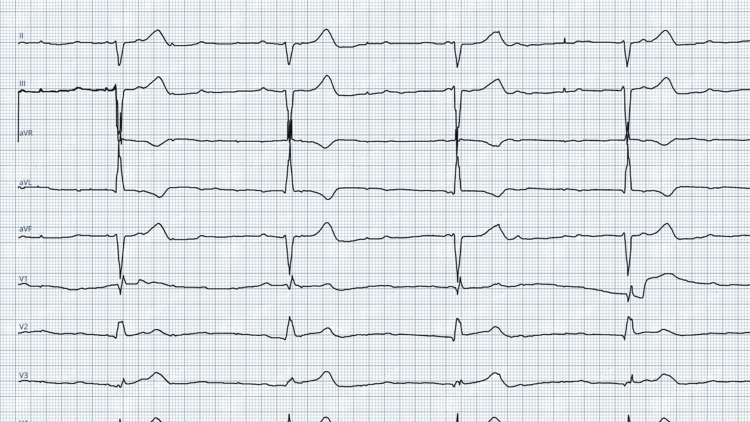
Electrocardiogram with failure to pace, absence of pacing spikes, and complete heart block with a wide-QRS escape.

**Figure 2:**
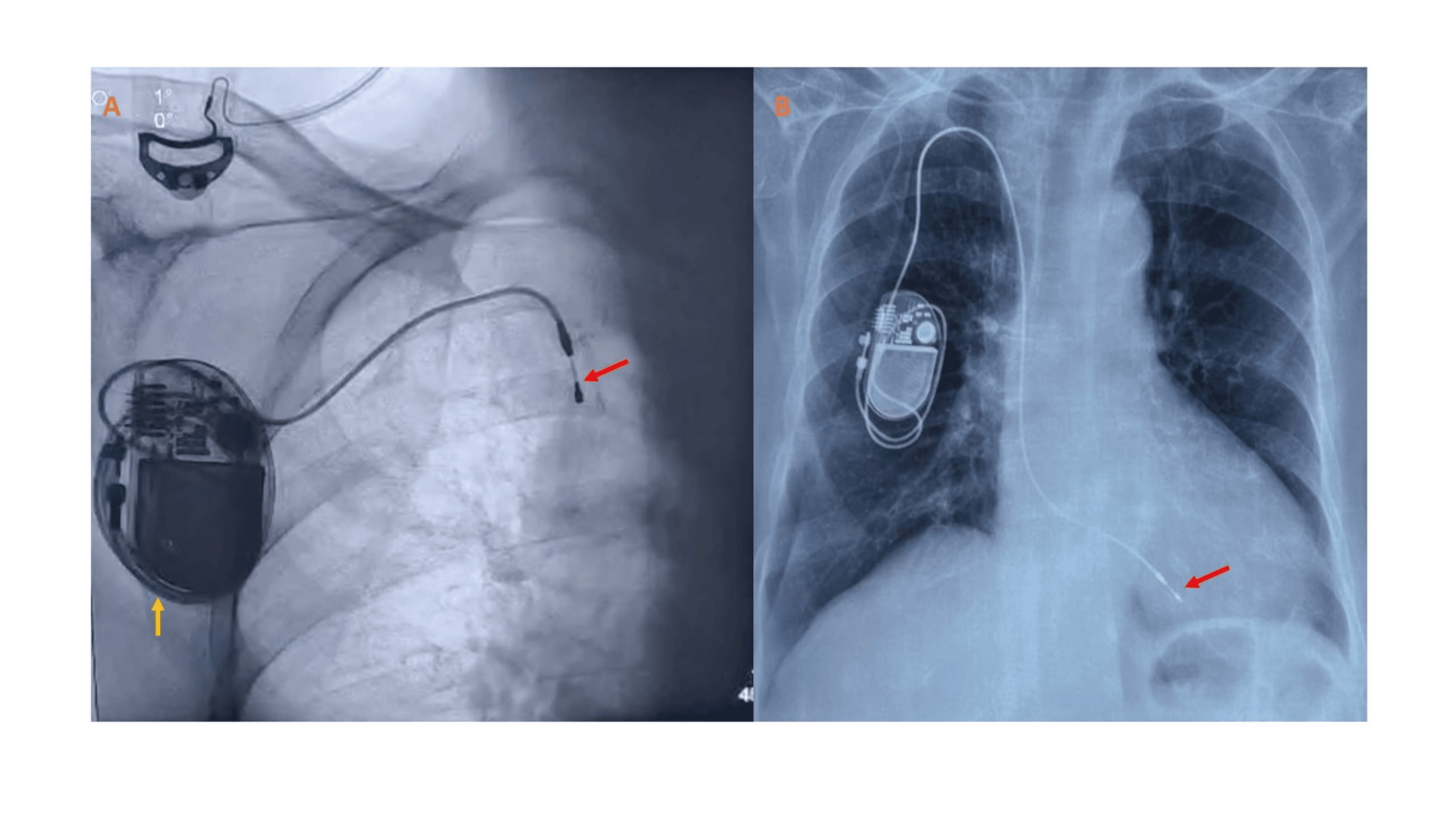
The distal part of the lead was pulled up into the superior vena cava, with the proximal lead coiled around the generator pocket (A). The final position of the lead after re-do procedure with an active fixation lead fixed to the lower part of the septum (B). RV lead tip lying in the SVC (red arrow) and pacemaker lead coiled around the pulse generator (yellow arrow). SVC: superior vena cava; RV: right ventricular

A 12-lead electrocardiogram showed complete heart block with failure to pace (escape rate 40 beats per minute) (Figure 1). Device interrogation showed the lead impedance to be 660 ohms (normal), but the sensed voltage and capture threshold could not be estimated. Cinefluoroscopy revealed a tined right ventricular lead which was coiled round the pulse generator, with its tip retracted to the superior vena cava (Figure 2, panel A). Two-dimensional transthoracic echocardiography and blood investigations were within normal limits. The prior cinefluoroscopy and chest radiograph (following the index procedure) were reviewed, which showed the tined ventricular lead in the right ventricular apex with an inadequate loop (Video 1). On comparison, the pulse generator appeared to have rotated around its transverse axis by around 270° in the current cinefluoroscopy with subsequent coiling of the lead, giving it the nomenclature of Reel syndrome.

**Video 1:** Cinefluoroscopy in the antero-posterior view showing the position of the pulse generator and ventricular lead in the right ventricular apex after the index procedure.

Based on the cinefluoroscopic findings and the risk factor profile (discussed below), she was diagnosed with pacemaker Reel syndrome. The other macro-dislodgement syndromes are Twiddler's and Ratchet syndromes. While the risk factors are similar, Twiddler's syndrome presents with rotation of the pulse generator about its longitudinal axis, causing the leads to twist and retract. Ratchet syndrome is characterized by retraction of the leads without rotation.

While Reel syndrome is mostly characterized by intact lead integrity and function, we still decided to replace the lead as it was tined. Under local anesthesia and mild sedation, the pacemaker pocket was re-explored. The lead was seen to be coiled on top of the pulse generator. Axillary venous access was taken, and an active fixation lead was placed at the lower right ventricular (RV) septum with a generous loop, while the old lead was removed (Figure 2, panel B; Video 2). The same pulse generator was connected and securely placed in the pocket. The procedure was uneventful, and the patient was discharged after suture removal and is doing well at six months of follow-up. Her pacemaker parameters revealed all pacing parameters to be within normal limits.

**Video 2:** Cinefluoroscopy in the antero-posterior view showing the right ventricular lead pulled up in the SVC and the lead being coiled up around the generator. SVC: superior vena cava

## Discussion

Complications following implantation of cardiac implantable electronic devices (CIEDs) are inevitable, especially with the greater use of more sophisticated and bulkier devices. These complications have been classified into access-related complications, lead-related complications, and generator-related complications [3,4].

Pacemaker lead dislodgement is a rare complication of permanent pacemaker (PPM) implantation. It is reported in nearly 2% of implants, especially in elderly, obese patients with large generator pockets [5]. Late (>6 weeks) complications of lead dislodgement are even more infrequent (0.2%) [6].

**Figure 3:**
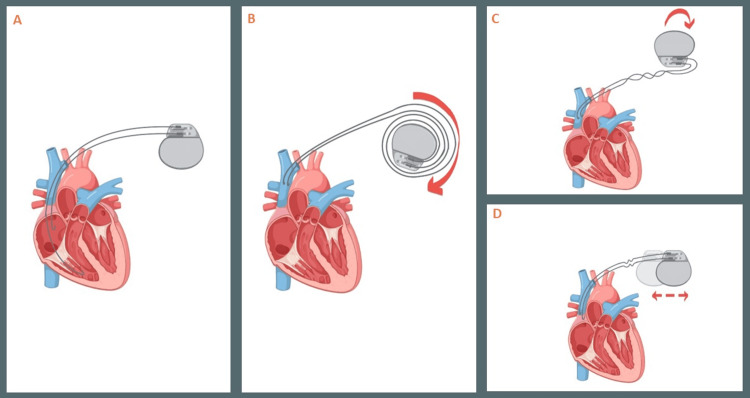
Pacemaker lead macro-dislodgement syndromes. Part A shows the normal position of the lead and the generator. Part B (pacemaker Reel phenomenon) shows the lead being pulled up with its distal end in the superior vena cava and the proximal end coiled around the generator. Part C (Twiddler's syndrome) reveals the lead being pulled up with coiling of the leads consistent with Twiddler's syndrome. Part D (Ratchet syndrome) reveals the leads being pulled up with the generator having moved from lateral to medial. This image was created by the author (Devesh Kumar) of this study using BioRender.com.

Twiddler's, Reel, and Ratchet syndrome are causes of pacing failure secondary to lead macro-dislodgement, defined by their lead and generator findings on radiographic images [7]. Well-known risk factors that predispose to these conditions include female gender, old age, obesity, cognitive impairment, spacious pockets, and, most importantly, loose lead and generator fixation at the time of the index procedure [8]. Reel syndrome is characterized by rotation of the generator on its transverse axis, much like the "reeling up of a coil" (Figure 3, panels A-D). This results in subsequent lead dislodgement, often in the absence of significant lead damage [7,9]. Twiddler's syndrome, in contrast, results in lead dislodgement and braiding due to generator rotation on its long axis caused by either intentional or unintentional external device manipulation. This condition frequently requires lead replacement because of the effect of torsional force and friction on lead integrity. Lastly, Ratchet syndrome is caused by "sliding" of the generator back and forth, laterally along the frontal plane, resulting in lead displacement [10].

Pacemaker lead dislodgement may have a plethora of varied presentations (Table 1). While it may present with failure to pace and subsequent conduction defects, there may be many atypical presentations. Patients may present with extra-cardiac manifestations posing a diagnostic challenge. Stimulation of extra-cardiac structures by the dislodged lead often results in regular contractions of muscles at the same rate as the pacemaker backup rate. The lead may stimulate the phrenic nerve, resulting in diaphragmatic contractions which present as abnormal abdominal pulsations [11]. Most of these patients had to undergo a re-do procedure with lead repositioning, as highlighted in Table 1. In our patient, we changed the lead as it was tined, and an active fixation lead would provide more secure placement in our view. Due to the diverse manifestations of lead dislodgement, a stepwise approach is crucial for all patients with cardiac or neuromuscular presentation and a history of PPM insertion. A structured approach consisting of electrocardiography, chest radiograph, cardiac monitoring, and pacemaker interrogation is indispensable when dealing with lead dislodgement [12].

**Table 1:** Summary of previously reported pacemaker Reel syndrome cases, their risk factors and their management. PPM: permanent pacemaker; AICD: automated implantable cardioverter defibrillator

Studies	Presentation	Risk factors	Management
Bellinge et al. (2021) [13]	Abdominal wall pulsations, abdominal pain, and lower limb jerking 3 months following PPM insertion.	Elderly lady, obese, with dementia	Re-do procedure with lead re-positioning
Rai et al. (2021) [14]	Complete heart block with syncope	Elderly lady, obese, re-do procedure with lax subcutaneous tissue	Re-do procedure with lead re-positioning
Mohammad et al. (2018) [1]	Multiple inappropriate shocks in a patient with AICD	Elderly lady	Re-do procedure with lead re-positioning
Alvarez-Acosta et al. (2014) [9]	Complete heart block with syncope	Elderly lady	Re-do procedure with lead re-positioning
Singh et al. (2018) [15]	Complete heart block with syncope	Elderly lady	Re-do procedure with lead re-positioning
Carnero-Varo et al. (1999) [7]	Complete heart block with pre-syncope	Elderly male	Re-do procedure with lead re-positioning

Steps to prevent pacemaker lead dislodgement are therefore of paramount significance to cardiologists. Important tips include avoiding spacious pockets, subpectoral implantation, and securing leads and the generator with non-absorbable sutures via an anchoring stitch technique. Lastly, when the risk of macro-dislodgement is high, the pulse generator may be anchored to the pectoral muscle using non-absorbable sutures, and the electrode can be secured via the use of active fixation leads. A firm "tug" at the time of PPM lead securing ensures good fixation of the lead [13].

Other important precautions include education about potential complications of PPM insertion, with a focus on the avoidance of device manipulation. Using an arm sling may assist in avoiding any device manipulation by limiting arm movement [14].

## Conclusions

Utmost care is required during pacemaker implantation in patients at higher risk of lead dislodgement, such as the elderly and obese patients, and females with dementia. Patients with a PPM who present with failure to pace, undifferentiated neuromuscular complaints, or syncope should be evaluated to rule out lead dislodgement. They should undergo an electrocardiogram, chest radiograph, and cardiac monitoring until a pacemaker interrogation can be performed. In patients with a pacemaker, Reel lead repositioning is generally adequate, whereas most patients with Twiddler's syndrome warrant lead replacement.
